# LiSA: an assisted literature search pipeline for detecting serious adverse drug events with deep learning

**DOI:** 10.1186/s12911-022-02085-0

**Published:** 2022-12-22

**Authors:** Vincent Martenot, Valentin Masdeu, Jean Cupe, Faustine Gehin, Margot Blanchon, Julien Dauriat, Alexander Horst, Michael Renaudin, Philippe Girard, Jean-Daniel Zucker

**Affiliations:** 1 Quinten, 8 rue Vernier, 75017 Paris, France; 2 Swiss Agency for Therapeutic Products, Swissmedic, Hallerstrasse 7, 3012 Bern, Switzerland; 3 UMMISCO, Sorbonne University, IRD, Bondy, France

**Keywords:** Adverse drug events, Assisted literature review, Deep Learning, NLP

## Abstract

**Introduction:**

Detecting safety signals attributed to a drug in scientific literature is a fundamental issue in pharmacovigilance. The constant increase in the volume of publications requires the automation of this tedious task, in order to find and extract relevant articles from the pack. This task is critical, as serious Adverse Drug Reactions (ADRs) still account for a large number of hospital admissions each year.

**Objectives:**

The aim of this study is to develop an augmented intelligence methodology for automatically identifying relevant publications mentioning an established link between a Drug and a Serious Adverse Event, according to the European Medicines Agency (EMA) definition of seriousness.

**Methods:**

The proposed pipeline, called LiSA (for Literature Search Application), is based on three independent deep learning models supporting a precise detection of safety signals in the biomedical literature. By combining a Bidirectional Encoder Representations from Transformers (BERT) algorithms and a modular architecture, the pipeline achieves a precision of 0.81 and a recall of 0.89 at sentences level in articles extracted from PubMed (either abstract or full-text). We also measured that by using LiSA, a medical reviewer increases by a factor of 2.5 the number of relevant documents it can collect and evaluate compared to a simple keyword search. In the interest of re-usability, emphasis was placed on building a modular pipeline allowing the insertion of other NLP modules to enrich the results provided by the system, and extend it to other use cases. In addition, a lightweight visualization tool was developed to analyze and monitor safety signal results.

**Conclusions:**

Overall, the generic pipeline and the visualization tool proposed in this article allows for efficient and accurate monitoring of serious adverse drug reactions from the literature and can easily be adapted to similar pharmacovigilance use cases. To facilitate reproducibility and benefit other research studies, we also shared a first benchmark dataset for Serious Adverse Drug Events detection.

## Introduction

The development of a drug is a long road that can take several years. This journey involves several requests for approval with regulatory authorities, whether to start clinical trials, to actually market the drug or to modify some of the claims. Throughout these approval processes, the regulator, that carries out a public safety mission, must ensure that no prior safety signal about the drug is known at the time or after the authorization is granted. This task requires the regulator to review and monitor both biomedical literature and surveillance reports. More specifically, medical reviewers have to identify portions of text mentioning an explicit association between a drug and a serious ADR. According to the EMA, a serious adverse event is “any untoward medical occurrence that at any dose:

- results in death,
- is life-threatening,
- requires inpatient hospitalisation or prolongation of existing hospitalisation,
- results in persistent or significant disability/incapacity, or
- is a congenital anomaly/birth defect.”

It should be distinguished from what is called an Important Medical Event (IME) where the outcome might not fall into one of these 5 categories. For example, in the sentence *There was one treatment-related death due to myositis in the pembrolizumab group.*, the serious outcome (death) is clearly associated with the drug (pembrolizumab) through the expression (treatment-related). Conversely, in *We observed Rivaroxaban-induced rash in* $60\%$ *of the patients*, the side effect mentioned cannot be qualified as serious. As such, it would be regarded as a safety issue by the regulator. Meanwhile, the tremendous increase of publication volume, and the number of treatments that require authorization in a limited time frame make it practically impossible for medical reviewers to review all documents exhaustively. Consequently, critical safety-related information can be missed when applying a human-only process.

Even though many publications have focused on literature review assistance [1–3] or on the detection of relationship between drug and ADR [4–8], only two have proposed approaches to tackle the detection of seriousness [9, 10]. Meanwhile, in the first publication, the targeted documents are FAERS reports which differ from biomedical literature in terms of syntax and vocabulary. The second one, thus tested on biomedical corpus, does not provide any kind of relationship between a drug and an adverse event.

In this paper, we present LiSA (Literature Search Application), an AI-based system designed to assist medical reviewers in their market surveillance by automatically screening the biomedical literature to detect safety signals.

LiSA was designed to enable medical reviewers to monitor the publication of articles related to potential safety signals on medical treatments or medicines. More specifically, it is able to identify, filter and rank publications mentioning an established relationship between a specified drug and one or several serious Adverse Events (SAE), i.e. severe Adverse Drug Reactions (SADR). To meet these goals, we propose 4 contributions to the problem of pharmacovigilance information retrieval from open data literature:

1. A deep learning pipeline for the identification of serious adverse events within biomedical literature based on Pub-Med. The performance achieved is respectively of 81.1$\%$ in precision and 88.6$\%$ in recall.
2. A visualization tool designed to allow biomedical expert to review and monitor the results provided by the pipeline for specific drugs.
3. A modular pipeline built on pre-existing and independent open source models (transformers) allowing flexibility of usage for related use-cases in pharmacovigilance. This approach also provides more explainability compared to a lone neural network algorithm. The pipeline, instead of creating a new neural network algorithm with very specific outputs, is composed of independent algorithms providing intermediate outputs. These outputs are then combined to build an efficient and performing system aiming at qualifying and extracting the information corresponding to the following questions:
  What are the monitored drugs and indication mentioned in the document?
  What are the sentences that mention an established relationship between a drug and an AE?
  What are the entities recognized as Drug or Adverse Event?
    The identification of relevant documents regarding seriousness drug adverse reaction signals is then performed on the basis of this information and meta data available in the data source (Ex: date, journal, type of publication, etc...).
4. A benchmark dataset for seriousness classification task based on PubMed literature sentences.

After a review of related work, we describe the LiSA pipeline architecture and provide a high-level performance analysis of the proposed solution.

## Related work

In most of the papers mentioned in this section, the focus is on Adverse Events (AE) detection and not on Adverse Drug Reaction (ADR), meaning that there is no specific detection of a drug associated with an adverse event. For the sake of clarity we will use, only in this part, the terms Adverse Drug Events (ADR) to indifferently designate AE or ADR.

Adverse Drug Reaction detection plays a key role in drug-safety surveillance and has motivated the creation of various monitoring systems or databases. The FAERS [11] reporting system and Medwatch [12], a medical product for safety reporting, are the current official solutions provided by the FDA. Meanwhile, these tools are only based on declarative reports and not on systematic analysis of the biomedical literature or any web-based source to identify potential ADRs. Several solutions have been proposed to perform biomedical literature monitoring in order to identify, filter and rank papers related to a specific domain or medical concept. For example, ASE [13] demonstrates the value of reference management, statistics, natural language summarizing to interactively select key papers. STELLAR [3] leverages data mining techniques to help researcher to identify, rank and recommend reference papers for a specific literature review. More recently, [1] proposed ASReview, an efficient active learning based-tool to perform systematic literature review and meta-analysis.

As per today, only a small number of literature review systems relate to adverse drug reactions detection. Among them, the PV-OWL tool [2] was built to link different databases to obtain novel safety indicators (FAERS, PubMed, social media...). The semi-automated pipeline published by [14] supports extracting ADR pairs from adverse events databases using statistical BPCNN algorithm for Natural Language Processing. Among other classical approaches commonly used in NLP, distributional semantics based on patterns of ADR co-reporting [15], Hidden Markov Models [16] or disproportionality analysis (DPA) [17] were already attempted to perform ADR detection. In 2012, Gurulingappa, Harsha et al. published an open-source reference dataset and developed a dictionary-based algorithm for extraction of adverse drug events in PubMed literature [18, 19]. Following the significant advances in natural language processing with deep learning, more recent publications have exploited these technologies to improve safety signal detection. Several works perform ADR detection and extraction on social networks (e.g. Twitter) or on drugs review platforms like *Drugs*.*com* using deep learning techniques [4–8].

However, there is a lack of studies aimed at predicting the *seriousness* of adverse events or any other type of qualification. The seriousness of an adverse event is nevertheless critical since it will decide whether or not to trigger actions from the safety surveillance agencies. We only found two publications related to this specific topic. The first one from [9] is based on FAERS report and does not treat biomedical literature. On the contrary, the second provides a robust approach to detect, extract and categorize serious adverse events [10]. The study relies on three different deep learning algorithms for seriousness classification, seriousness categorization and seriousness annotation. Performance is evaluated on three datasets among which one is built on biomedical literature. Like the latter study from [10], which will also be used as the primary basis for performance evaluation, LiSA is capable of qualifying potential severity but differs in its ability to detect and extract adverse drug reaction entities and classify documents for display in a literature search tool interface.

## The LiSA pipeline description

**Fig. 1:**
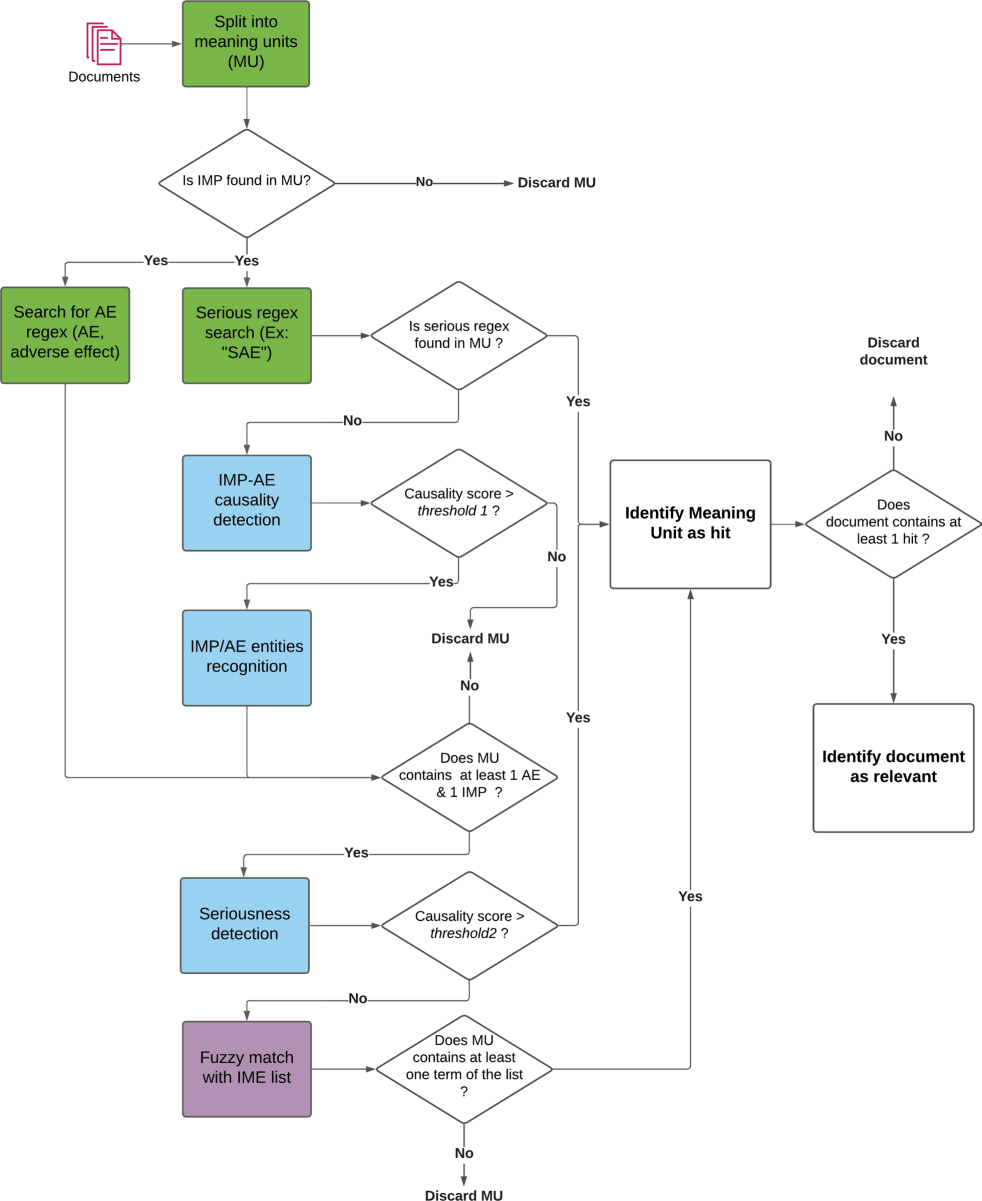
Decision diagram of the document processing pipeline. Green color boxes represent regex-based algorithms, blue color boxes represent deep learning based algorithms and purple color boxes fuzzy-matching based algorithms

The architecture described in this section is the final result of a sequence of iterations aimed at improving the overall performance to maintain a satisfactory balance between precision and recall (more details are available in the “Results and discussion” sect.). The objective of the following steps is to identify and extract relevant information in documents (drug names, Adverse Events, association between drug and AE, seriousness,...) to be used for the final ranking and filtering of articles. The document processing pipeline is described in Fig. 1 below.

### Query definition and document collection

Most of the medical publications that mention adverse drug reaction are published and available through PubMed, a free archive of biomedical and life sciences journal literature and considered as a reference for biomedical publications. Some publications require a licensed access, but still provide a free version of the corresponding abstract. Therefore, PubMed was used as the main data source for literature monitoring to build the system. By construction, documents collection should be associated to a “query” which is composed of a combination search terms. A query contains a main drug, an optional second drug and optional indication. Indication should be only approved indication to avoid the case where indication and adverse events are confused. Consequently, LiSA collects all articles available on PubMed published on the last six years^1^ associated to a query through the PubMed API. This timeframe was chosen as a good tradeoff between actuality of information and being sure not to miss a relevant signal that might have been reported a while ago. Only the main drug serves as keyword search to trigger the API. Other query terms (optional drug and indication) are only searched to tag the document if they are mentioned in it.

Drug and indication could be expressed under various synonyms in biomedical literature. To ensure the comprehensiveness of data collection, every drug and indication term is conjointly searched with all its synonyms based on open-source molecule and disease classification. For drug, we used the Chembl database [20], and for indications the MedDRA [21] hierarchy. Table 1 gives an example of a query definition.

**Table 1:** Example of a query definition used as input to LiSA

Drug 1 (main)	Drug 1 synonyms	Drug 2	Drug 2 synonyms [20]	Indications
DEBIO 1143	AT-406 D-1143 DEBIO-1143 IAP INHIBITOR AT-406 SM-406 Xevinapant	Cisplatin	NSC-131558 Platinol SM-406 Platinol-AQ	Squamous cell carcinoma of head and neck Squamous cell carcinoma of head and neck metastatic

### Document preprocessing

This section describes the methodology applied to preprocess documents into a suitable format for deep learning algorithms described below.

#### Data preparation

To structure the documents, we propose a standard architecture able to accommodate any type of collected documents or data sources and adapted to natural language processing algorithms. As a matter of fact, raw documents cannot be processed directly by transformers and achieve a satisfying performance [22]. They should be split into meaning units of limited number of tokens like sentence or short paragraphs. This process, called sentence tokenization, is performed with a pre-built algorithm (on common English language) from the package *nltk* and adapted with specific cases found in biomedical literature.

Structured data is then formatted into 3 different tables:

- *Documents table:* This table stores all the metadata and the full content of a document. This table contains one line per document.
- *Contents table:* This table stores only the content of a document but split in different sections or paragraphs based on the pre-defined structuration already available in the document (e.g.: abstract, methods, results, conclusions...). The contents available in figures captions or tables was not collected.
- *Meaning units table:* This table stores information at sentence level and is built from the contents table. A section or paragraph is split in different sentences and each sentence represents one line in this table. During the split, if a sentence is too short (between 4 to 10 words), it is concatenated with either the previous or the next one (only in case it is less than 20 words long) to reduce the risk of missing an AE-Drug relationship. These choices were applied for two reasons:
  - Concerning the maximum length of a meaning unit: BERT input size is limited to 512 tokens, which makes it impossible to use a whole article as input for prediction. Furthermore, it has been shown in the literature that BERT performs better on a limited number of tokens, therefore sentence as in input will be better than paragraph as in input.
  - Concerning the minimum length of a meaning unit: this decision was motivated by the empirical observation that in case of very short sentences, one information was actually present in the adjacent sentence. The threshold number of tokens was selected empirically and could be optimized in further work.

This generic structure has been designed to fit any type of document and serves as a basis for the visualisation tool presented in the “Visualisation interface” section.

#### Drug and indication search

The first filter applies to all meaning units found in collected documents and is based on a simple keyword search method. We use the Aho-corasick algorithm [23], an efficient dictionary-matching algorithm, to search for a drug term and associated synonyms in every meaning unit. Aho-corasick was used for its computation efficiency and because drug names have an invariant spelling in biomedical literature, there is thus no need to perform fuzzy-matching at this step. This association is then stored in the meaning units table. This step has a double objective:

- First, to isolate the meaning units associated with the drug of interest (since LiSA is built to monitor serious adverse events associated with a defined drug).
- Second, to reduce the number of meaning units to be used as input for the downstream deep learning modules that are more computationally intensive.

At the same time, a second keyword search is applied to identify *mentions of therapeutic indications* in historical documents. Unlike drug search, indication search is only performed at document level and is used to provide a clue of whether the document discusses about a drug aiming at treating a specific indication. The detection of an established relationship between a molecule and a disease is not performed in this pipeline. This task would be part of a possible improvement. The indication of interest are defined by biomedical reviewers and enriched with associated synonyms using the MedDRA hierarchy. Meanwhile, unlike drug names, indication terms are frequently composed of multiple tokens, which are not always expressed with the exact same form in the literature. For example the MedDRA indication “B-cell chronic lymphocytic leukaemia” could be found as “B-cell lymphocytic leukaemia” or “lymphocytic leukaemia of B-cell” in published articles. Therefore a simple expression search will most likely miss some expressions associated to the same indication. To overcome this problem, we built a fuzzy-matching algorithm allowing permuted and incomplete expression of an indication to be found in the text, which creates a list of expressions on the basis of a root indication. This list is composed of all permutations of the tokens contained in the root indication, with a random suppression of some of them to keep at least 2 tokens. All the expression of that list are then searched in the document, with the same Aho-corasick algorithm but allowing the presence of 20 characters between 2 consecutive tokens of the list. For example when searching for “B-cell lymphocytic leukaemia”, the expression “B-cell and C-cell lymphocytic leukaemia” will be accepted by this algorithm.

### Deep learning

**Fig. 2:**
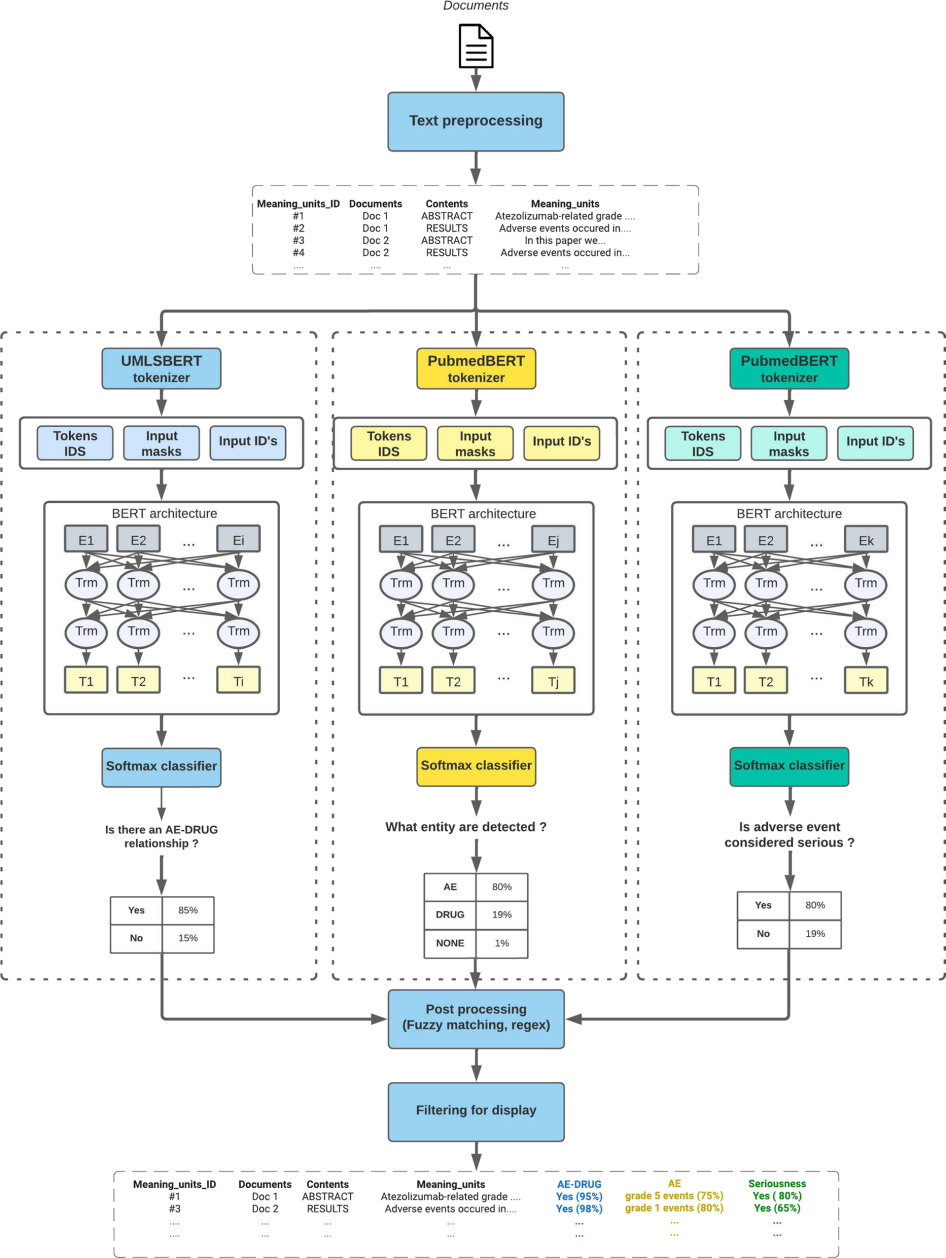
Schematized machine learning architecture of the LiSA pipeline and its three main modules. Unlike other post processing tasks, the serious regex search task is performed before deep learning inference and is not represented on the schema

The three main AI modules presented in this section are the core of LiSA. They correspond to 3 different NLP tasks which are computed in parallel for all sentences containing a monitored drug (as described in the next section). Once calculated and stored in the database the different information are used to filter and qualify the hit sentences and relevant documents as depicted in Fig. 2. Details about the different pre-trained algorithms and their respective performance are provided in Table 3.

#### Drug-AE relationship classification

To assess the association between a drug and an AE, we chose to rely on state of the art deep learning algorithms with attention-based mechanism (BERT). This family of algorithms is trained on very large corpora to build contextual embeddings and has been shown to perform extremely well in highly context-dependent prediction tasks, such as AE detection. The presence of a drug-AE causality relationship within a sentence was predicted with a two class (“has causality”, “has no causality”) sentence classifier, as defined in the ADE-Corpus-V2 dataset [18] used for training. This dataset contains more than 20 000 sentences extracted from PubMed and pre-labelled for drug-AE causality classification. In particular, the two classes are defined without prior knowledge of the entities corresponding to drug and ADRs. The ADE-Corpus-V2 dataset was split into training, validation, and testing sets with the ratio of 8:1:1 and used to fine-tune several pre-trained algorithms and to select the most accurate one.

In order to further increase prediction performance, we performed manual data augmentation based on badly predicted observations of ADE-corpus-V2. Typical treated case are sentences including a negation form, containing an unspecified adverse effect (“AEs”, “TRAEs”, “Serious adverse effects”) or related to specific lexical fields. The score threshold to predict a sentence as positive was chosen at 0.2. This value offers the highest possible recall and keep precision higher than 0.9 (threshold determination was manually performed based on a precision-recall curve) In the production version of LiSA, every meaning units predicted class and score are stored in the meaning units table.

#### Named Entity Recognition (NER)

LiSA is also supposed to identify the different entities found in a relevant document corresponding to a drug or an Adverse Drug Reaction. For this task, we used Named Entity Recognition (NER) pre-trained algorithms within the same family of algorithms built on BERT architecture. Using the same open source corpus, we fine-tuned and bench-marked several models for the task of identifying two different entities: drug and ADR.

The NER task was built as defined in the $ADE-corpus-V2$dataset [19]: find spans associated to 2 types of entities: DRUG and AE. No distinction was made between beginning, inside and outside tokens of a selected entity.

In the final pipeline, the entity detection is only applied on meaning units that successfully passed the drug-AE causality prediction with a score higher than the defined threshold (the standard threshold value 0.5 was used). This pre-filtering step was made to reduce the inference computation time. As for the previous step, detected entities and associated scores are stored in the meaning units database. The NER step was also applied after Drug-AE relationship classification since it reduces the computation time without major change in terms of performance. Inference time remains the most time-consuming task in the LiSA pipeline, which is critical for the system to be used in production.

#### Seriousness score prediction

According to the European Medicines Agency [24], an adverse event can be qualified of serious of the consecutive reaction to a treatment:

- results in death
- is life-threatening
- requires inpatient hospitalisation or prolongation of existing hospitalisation
- results in persistent or significant disability/incapacity
- is a congenital anomaly/birth defect.

This definition clearly underlines the fact that the seriousness of an ADR is measured according to the outcome that it produces, whose expression in a document, is here again, highly context-dependent. BERT-like architecture based on contextual embeddings is once more a very promising solution. The same training framework applied in the two previous NLP tasks was applied here. We fine-tuned several pre-trained models on a sentence classification task. Unlike common ADR detection, we did not found an open access dataset to train the seriousness detection algorithm. This problem was overcome by labelling 7776 sentences extracted from PubMed in three categories: “serious”, “important medical event”, “none” (a “serious” sentence being an “important medical event” sentence with a serious outcome). The labelling process was performed by medical reviewers and based on examples extracted from positive examples of the $ADE-corpus-V2$ dataset [18]. The third class “important medical event” was only added to have a more detailed labelled dataset for possible additional application in ADR detection. The ADR entities were not provided to the expert during annotation to force the annotator to take into account the full sentence and not only part of it (like extacted ADR) to make his decision. In addition, we performed data augmentation by semi-automatically building sentence examples to address some weaknesses of the algorithm in specific contexts or syntax (negation, cancer, etc..), that were also annotated by medical experts before being included in the training set. 917 sentences were used as a testing test and allowed to reach a performance at the state of the art. More concretely, this models yields a class and a score and is only calculated on meaning units that contains at least one drug entity and one ADR entity from the NER module.

#### Post-processing for performance improvement

Although the performances obtained by the previous pipeline on average matches the level reached in recent publications [10, 19] (more details in the “Results” Sect.), it appeared that some specific cases were relatively badly predicted. A typical encountered issue was a random detection of non specific adverse events corresponding to expressions like “AE”, “adverse effects”, “TRAEs”,... To address those issues, different strategies were implemented in addition to the improvement of the three previous deep learning algorithms by data augmentation.

The first strategy implemented was the use of regular expressions that by themselves indicate the presence of an adverse event in sentence. A few example of these are “side/adverse event(s)/effect(s)/reaction(s)” or “(TR)AE(s)”. The same method is applied to the case of non specific serious adverse events with regular expressions such as “serious adverse event(s)/effect(s)/reaction(s)”, “grade 4/5 reaction(s)” or “SAE(s)”. This double search is applied on all meaning units containing an drug of interest since they are computationally light.

The second strategy used is specifically designed to catch serious adverse outcomes based on a list of terms built together with biomedical experts. That list contains expressions of diseases or reactions that are always associated with a serious outcome (death, hospitalization, infirmity, congenital, life-threatening). This is for example the case for “pneumonia”, “ventricular fibrillation”, “intracranial bleeding”,“teratogenic effects”. The same fuzzy-matching approaches as the one described in the previous section is applied in this case, since we are considering multiple-tokens expressions. Unlike regular expressions search, the fuzzy-matching is only applied to meaning units that were rejected by the seriousness score algorithm to optimize the computation time.

### Document filtering and ranking

LiSA is built to provide a curated list of documents to the user, as well as the sentences where safety signals (called “hits”) are detected, and the recognized entities (drug and ADR). The decision process depicted in Fig. 1 is used to select and filter the documents to be finally displayed to the final user. It can appear counter-intuitive that the AE-drug relationship classification results are used before the entity recognition. This order showed the best performance and was selected after different experiments that are not detailed in this paper.

A rule-based system was also implemented to calculate a ranking score based on some information extracted from documents (sentence hit scores, number of hits per document,...). This score is then used by the user to rank the relevant papers in the visualisation interface.

### Visualisation interface

**Fig. 3:**
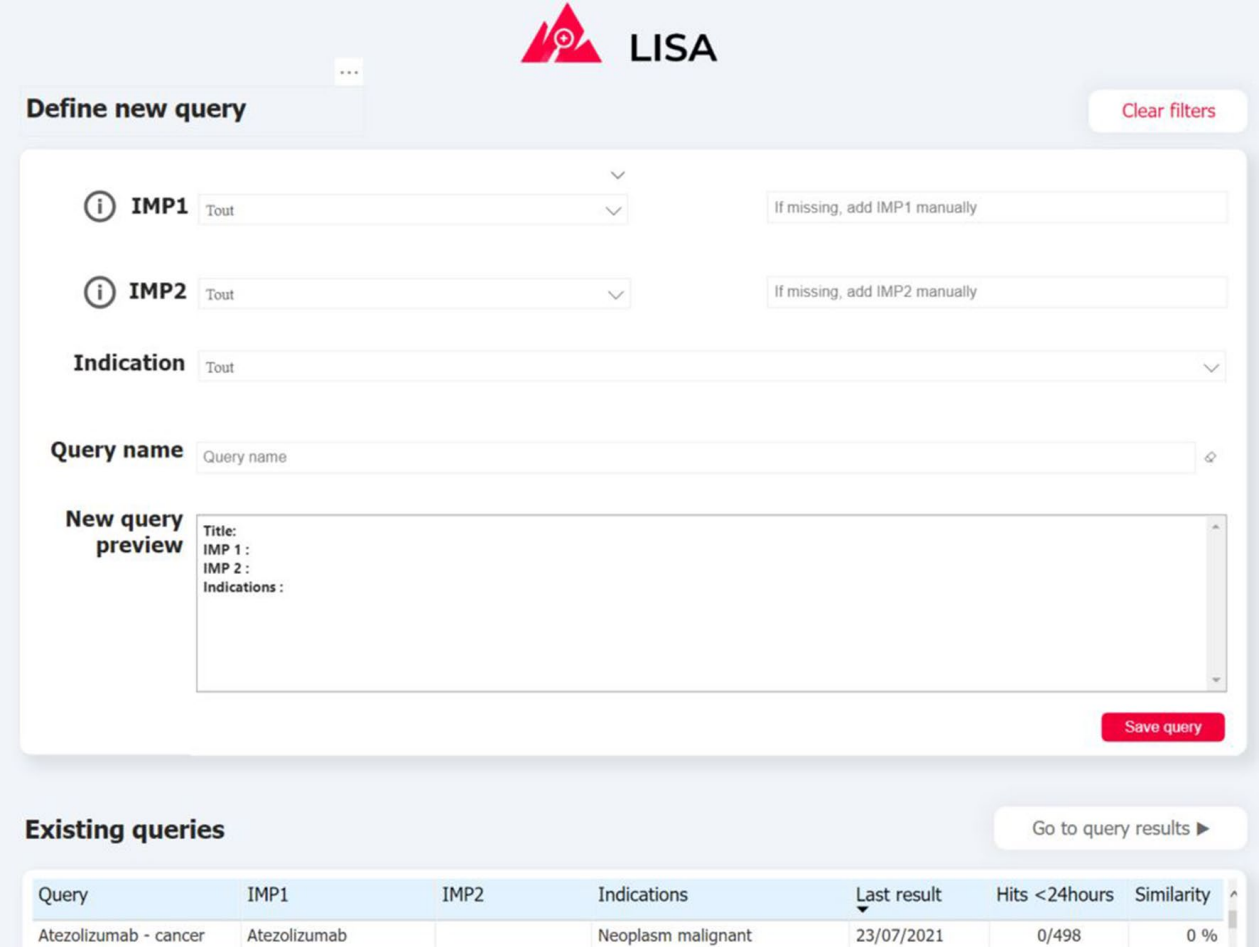
Screenshot of the “QUERY DEFINITION” tab of the interface. (1) Drug and indication dictionaries drop-down lists (2) Query preview (3) Summary of previously-created queries

Visualizing and exploring the results is key to ensure user adoption. Depending of the query definition, the pipeline can return a relatively large number of documents (volume of some example queries are provided on Fig. 5) indeed. In order to prevent users from being overwhelmed by a mass of articles to review, and in order for them to monitor results over time, we propose a simple exploration interface built with PowerBI, a powerful and cost-effective data visualisation tool. Captures of the two main interfaces are presented on Fig. 3,  4. First the QUERY DEFINITION interface allows a user to create or join search queries containing one or several search criteria, as defined above. Second, the RESULTS interface displays documents found in the literature, with at least one hit mentioning a serious drug adverse reaction. On the left side, a series of filtering options (publication date, indication found, AR Frequency, Route of administration, etc...) are available to help the user refine displayed results. These filters are fed by information already extracted by the pipeline, and by results from keyword searches performed by powerQuery (PowerBI’s data preparation engine). The results can be explored at a document/sentence level (high level results) showing only information down to the sentence and document, and at a more detailed level (detailed results) which includes ADR entities detected in the text.

**Fig. 4:**
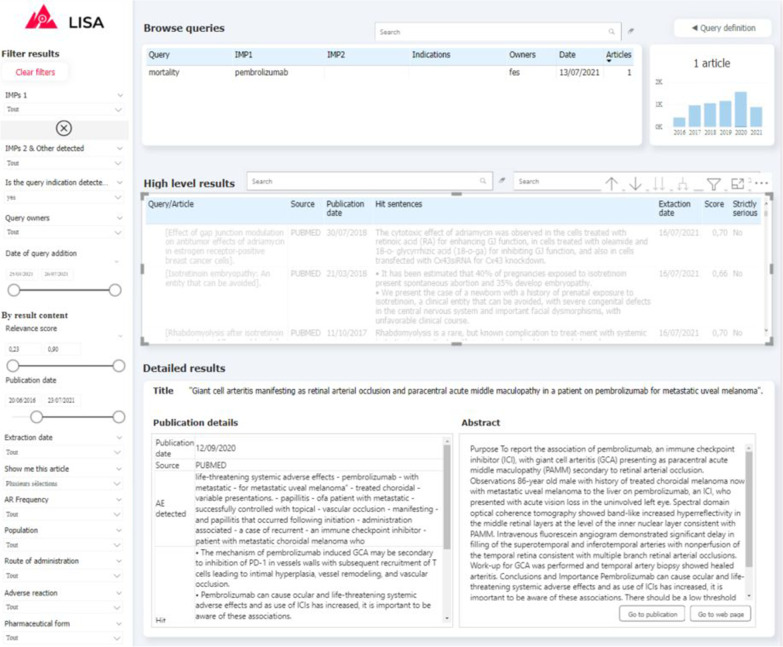
Screenshot of the “RESULTS” tab of the visualisation interface. (1) Query browser to select a set of results (2) Filtering tab to refine query results (3) High-level results table containing general information about results associated to the selected queries (4) Detailed results which provide additional information for every article selected in the high-level results table (5) Histogram of results volume of publication by year for the selected queries

## Results and discussion

The following section is dedicated to:

- Describing the obtained results and justify the need for the use of a new benchmark dataset for evaluating the task of serious ADR detection.
- Discussing the limits of the current pipeline and pave the way for future work.

### Results

Performance assessment was performed with two strategies:

- Evaluate the results based on a train/test approach on different datasets for different tasks. The performance of the tested models is displayed in Table 3.
- Evaluate the performance of LiSA from the perspective of medical reviewers (end users).

#### Implementation details

For individual NLP tasks evaluation, we used a specific test dataset for each task. This test set was created by selecting 10$\%$ of available labeled data that remained unseen by the algorithm. For AE-drug relationship classification as well as NER, we used the ADE-corpus-v2 dataset. For seriousness classification, the test set was carved out of the manually labelled dataset mentioned in subsection. Training was systematically performed with a learning rate of 3E-5, using the Adam optimizer and a batch size equal to 16. The pre-trained language model used in the evaluation are detailed in Table 2.

**Table 2:** The different pre-trained language models considered in the evaluation, their version in the HuggingFace repository and the type of pre-training

Pre-trained Language model Version	Corpus	Pre-training
UMLSBert_ENG [25]	Pubmed + UMLS	Continual pretraining
		+ weight adjustement
biobert-base-cased-v1.1 [26]	PubMEd	Continual pretraining
bluebert_pubmed_uncased	Pubmed + MIMIC III notes	Continual pretraining
_L-12_H-768_A-12 [27]		
scibert_scivocab_uncased [28]	Semantic Scholar	From scratch
Bio_clinicalBERT [29]	MIMIC III notes	Continual pretraining
BERT-base-uncased [30]	Wikipedia	From scratch
BiomedNLP-PubMedBERT-base-	Pubmed	From scratch
uncased-abstract-fulltext [31]		

#### Evaluation metrics

We choose to first evaluate the performance separately at task level and select the best performing algorithm according to results displayed in Table 3. Meanwhile, a good performance of each independent algorithm does not necessary imply a good performance of the whole pipeline. This could especially be the case if the decision process that narrows down the scope of relevant sentences with successive filters becomes too restrictive. In addition to that, the performance of each independent algorithm is calculated at the meaning units level and not at the document level, which is a more representative metric for the intended use-case of LiSA. Nonetheless, performance evaluation at document level is difficult since it requires to find a sample corpus of relevant publications in the literature. That sample should have the same ratio of relevant and irrelevant documents available in PubMed. However it is almost impossible to estimate that ratio unless going through hundreds of articles for every single drug.

**Table 3:** Measured Precision, Recall and F1-score performances on the three NLP tasks implemented in the pipeline on test sets

	AE-Drug relationship classification			Named Entity Recognition			Seriousness classification		
	P	R	F1	P	R	F1	P	R	F1
UMLSBERT	0.94	**0.93**	**0.93**	0.94	**0.96**	0.95	0.89	0.87	0.88
bioBERT	0.91	**0.93**	0.92	0.96	0.95	0.95	0.89	0.90	**0.89**
blueBERT	0.93	0.89	0.91	0.96	0.93	0.94	0.73	0.83	0.78
sciBERT	0.94	0.92	**0.93**	0.95	0.95	0.95	**0.92**	0.81	0.86
Bio_ClinicalBERT	0.94	0.92	**0.93**	**0.97**	0.92	0.94	0.68	**0.93**	0.79
BERT	0.90	0.89	0.90	0.95	0.92	0.93	0.76	0.74	0.75
PubMedBERT	**0.95**	0.90	0.92	0.96	0.95	**0.96**	0.87	0.91	**0.89**

The best value per column is in bold. ThFor the drug/AE entity recognition task, the displayed metrics only concern the AE class. The best model was selected for each task, PubMedBERT for NER and seriousness classification, UMLSBERT for AE-Drug relationship classification

Instead, we propose two methods to measure the global performance of the pipeline. First, we calculate the precision and recall at sentence level only, with a sample dataset extracted from PubMed. Second, we propose to evaluate LiSA with a simple keyword search-based method to perform safety monitoring literature review.

#### Dataset-based performance evaluation

**Sentence level evaluation of LiSA**

**Fig. 5:**
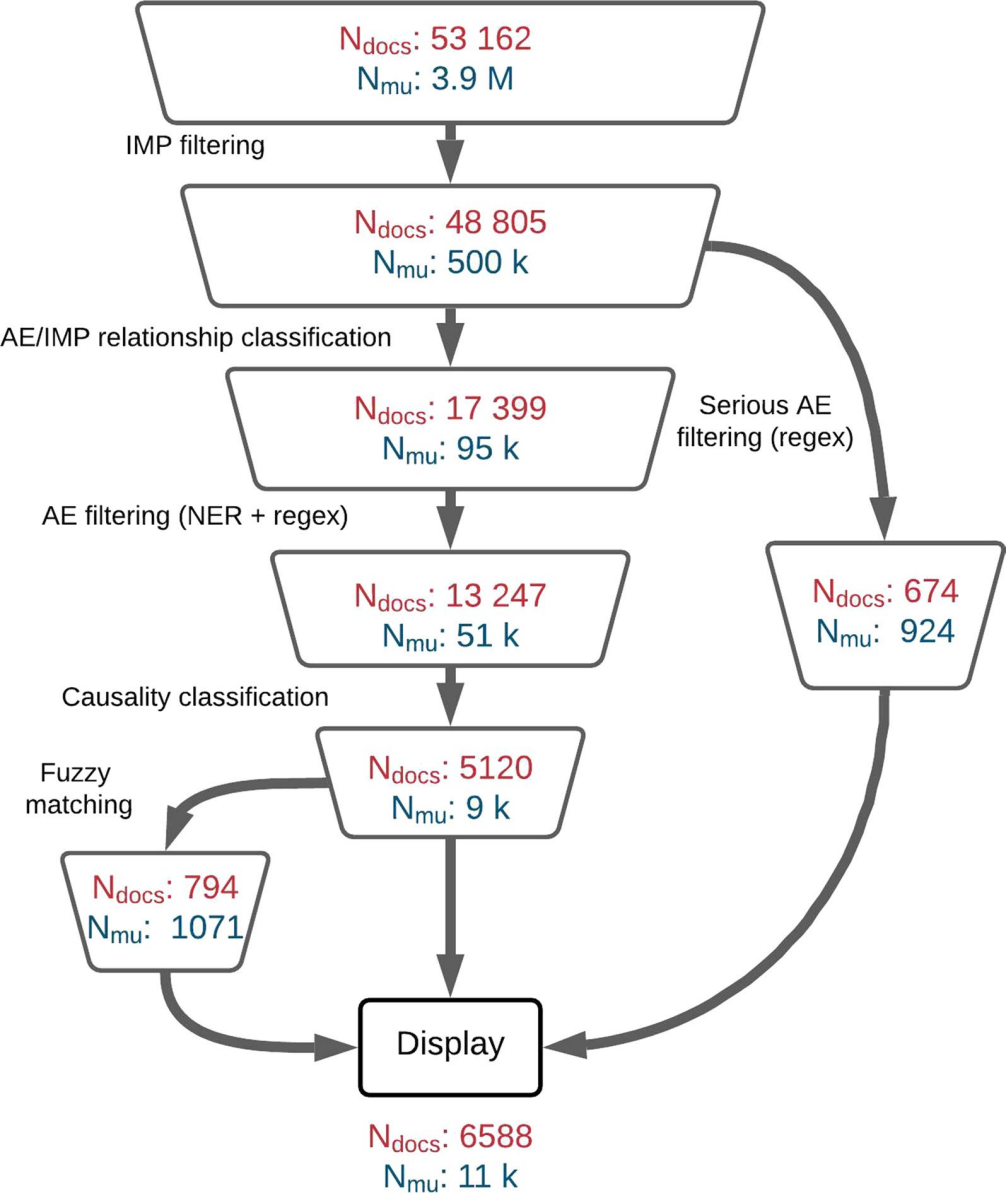
Volume of documents ($N_{docs}$) and meaning units ($N_{mu}$) after all decision steps in the LiSA pipeline. The volume corresponds to the documents collected with the drug list available in “Appendix”

To assess the performance at sentence level, we chose to use the classic performance metric for binary classification: precision, recall and f1-score. To calculate those metrics, we retrieved all documents associated with a list of drugs, as described in “The LiSA pipeline description” section. The list of selected drugs was selected to demonstrate how LiSA performs with new preparations, named with labcodes, and with established tradenames and comprise compounds for which certain signals were known to the experts in order to check whether they had been found accordingly (the list is available in “Appendix”). All documents were then fed into the LiSA pipeline to detect all positives sentences (hits) and their parent articles. The volume of documents and meaning units after every successive filter is available in Fig. 5.

In the absence of a benchmark dataset to evaluate the performance of serious ADR detection, we created the SADR dataset with the help of medical reviewers with the following procedure. We first collected all documents freely available on Pubmed that contains a drug in the list available in “Appendix ”, and only kept the sentences that explicitly contains one of the drugs (since its absence would inevitably make the sentence irrelevant). These sentences were passed to the pipeline to get a prediction regarding the presence of a serious ADR. Then we asked medical experts to review the sentences and check whether the prediction was correct or not. In total, 1231 sentences from 988 unique documents were analyzed, among which 275 are abstracts only and 713 also provide main text. Tables and figures were not analyzed, as well as references. In that sample, LiSA reached a performance of 88.6 $\%$ in recall, 81.1 $\%$ in precision and 84,7$\%$ in F1-score. We observed better results on abstracts sentences with 89.7 $\%$ in recall, 81.4 $\%$ in precision and 85.3$\%$ in F1-score than on documents other parts (TITLE, INTRO, METHODS, RESULTS, DISCUSS, CASE, CONCL). More details is provided in Table 4.

**Table 4:** Measured performances at sentence level across publications section types

Section	Recall	Precision	F1-score	Volume
TITLE	0.91	0.91	0.91	34
ABSTRACT	0.81	0.90	0.85	448
INTRO	0.80	0.90	0.85	226
METHODS	1.00	0.88	0.93	88
RESULTS	0.85	0.85	0.85	203
DISCUSS	0.75	0.86	0.80	183
CASE	0.67	1.00	0.80	25
CONCL	0.86	1.00	0.92	24

The achieved performance makes LiSA a state of the art system in terms of safety signal detection for the use-case considered in as much as it is closed to the performance obtained in [10]. Meanwhile, the task evaluated in this paper differs from the case of LiSA. Especially, there is no mention of a drug-AE relationship classification task. In addition there is no code available neither benchmark dataset from [10] that could have been used for direct comparison. For benchmark purpose, we provide the test dataset used to assess LiSA’s performance at sentence level, in supplementary materials.

The performance is higher for recall than for precision. This was designed on purpose, since there is a stronger need to not miss safety signals publication than achieving a higher precision. This optimization towards recall was especially enabled by the additional post processing modules described previously.

As far as the total number of collected documents and meaning units is concerned, as displayed in Fig. 5, LiSA is able to perform a very imbalanced prediction task with a high precision. Indeed with more than 53k documents and 3.8 millions meaning units to filter, there are only $0.2\%$ of meaning units that should be considered as relevant, for about $10\%$ of all collected documents.

**Document level evaluation of LiSA**

As mentioned before, evaluating the performance at document level is quite challenging. We can calculate the precision using the benchmark dataset available in “Appendix”. Over the 988 documents contained in the benchmark dataset, we found a precision of $78.5\%$.

Meanwhile, we are not in capacity to provide a good estimation of LiSA document recall. For that purpose, we should be able to measure to which extent the system is able to avoid missing relevant articles in the literature, which would require to label a corpus of at least a few thousand documents (which corresponds to about 80 000 sentences in total). This is an extremely time consuming task and is not immune to potential bias during the document selection phase to build the sample corpus.

#### User-based performance evaluation

To further assess the ability of LiSA to perform an efficient and comprehensive literature review on safety issues, we compare the results obtained by an expert medical reviewer using LiSA and using a simple keyword based search on PubMed. This type of evaluation is common in other systems for assisted literature [32].

For that purpose, we selected one drug, chosen for its relatively low number of associated papers found in the literature, making an exhaustive safety survey difficult. The goal is to compare the number of relevant articles that a manual search would yield to a LiSA-assisted search. On the one hand, a medical reviewer was asked to perform keyword search on PubMed with the expression “drug” + “serious adverse events” to review as much papers as possible within 2 h and retrieve the relevant papers and sentence hits only relatively to the presence of a serious adverse event. Some examples of queries used for this work are “sildenafil adverse events”, “emtricitacine serious adverse effects”. On the other hand, a second medical reviewer was asked to do the same literature review based on LiSA interface, within the same time frame. We also performed the same work for a drug notoriously known for its serious adverse drug effects: Azetolizumab. Due to the large number of papers mentioning serious ADRs in the literature (a few hundreds), the comparative performance between LiSA and a manual search is not significant. Time frame was limited because LiSA aims at speeding up drug monitoring process. Providing unlimited time to medical reviewer is not realistic regarding their daily work. In addition, the two reviews were performed by a different reviewers in order to ensure that the results of the second review will not be influenced by the first one if the same reviewer was doing both of them. Inter-rater Reliability between reviewers was measured on other molecules and was superior to 95

For a survey based on the drugs “Emtricitabine” and “Aflibercept”, the results achieved were as follows:

**Emtricitabine:**

- 7 articles were found with the keyword-based search
- 18 articles were found with the LiSA-assisted search.

**Aflibercept:**

- 8 articles were found with the keyword-based search
- 17 articles were found with the LiSA-assisted search.

The use of LiSA therefore makes it possible to largely increase the volume of relevant papers found during a defined search time (by a factor 2.5), especially when serious ADRs mentions are rare in the literature.

### Discussion

#### Comparison with state of the art models

The comparative analysis of pre-trained language models has shown different behaviors depending on the task:

- for AE-Drug relationship, no major differences were observed between the 7 selected models. This is most probably linked to the nature itself of the task which consists in detecting an association/causality relationship. This will not depends on specific biomedical vocabulary but rather on grammatical forms used to link a drug to an adverse event. This is probably why non-biomedical models like BERT and sciBERT also obtained good results. UMLSBERT provided the best baseline in terms of F1-score and was then selected.
- For Named Entity Recognition, the ability of a model to properly identify entities highly depends on the vocabulary learned by the model. On Table 3, the F1-score levels largely hide subtle differences in performance for specific biomedical sub-domain. Especially, we observed that UMLSBERT and PubMedBERT performed better on text related to oncology where there is a subtle difference between Adverse Events and drug effects related to drug mechanisms (that could be destructive). The specific pre-training of these algorithms might explain their superiority over other models used in the benchmark. We choose PubMedBERT as the best performing model.
- For seriousness classification, the vocabulary mastered by the model also highly matters. Indeed, many serious adverse events expressed with technical terms are by essence considered as serious (Stevens Johnson Syndrom, Rhabdomyolysis, Agranulocytosis...) and are better captured with specialized models like PubMedBERT, BioBERT and UMLSBERT. PubMedBERT was selected in this case.

The lack of extensive work on seriousness detection of Adverse Drug Reactions in the literature makes the comparison difficult to perform. In addition to that, the only publication [10] that tackles the problem does not provide any code implementation. Thus, apart from re-implementing the solution, there is no possibility to compare our algorithm with the one of this publication. Meanwhile, on a corpus extracted from Medical Literature, our pipeline reached a higher performance up to 0.81 in precision and 0.88 in recall (respectively compared to 0.83 and 0.82 [10]. Even if the dataset are not strictly comparable, we can conclude that our pipeline reached a state of the art performance on the specific task of seriousness classification.

Besides, the calculated overall performance of the pipeline at document level relies on a reduced number of documents (988). The statistical significance of the conclusion might be arguable since we cannot cover all the variety of semantic fields available in PubMed. Meanwhile, we believe that the global performance remains valid, especially since it is added to the already good performance achieved at sentence level, and calculated over a larger volume of examples.

#### Pipeline flexibility and portability

One important objective of the study was to build a system with a flexible architecture to enable the use of the pipeline on related use cases. For example, we could replace the seriousness classification by seriousness categorization (Death, Hospitalization, IME, Disability, Congenital anomaly [10]) or adverse events grade classification (Grade 1 to 5). This adjustment would of course require to train a new algorithm (for seriousness categories or adverse events grades classification) but with no impact of the 2 other modules. This is made possible by the independence of the three algorithms, them not being chained. They can then perform inference on the same type of input (a sentence containing at least one monitored drug). This approach is likely to introduce an overlap between the 3 NLP tasks that could be criticized, but allows a full flexibility in the combination of their outputs to build the required decision process.

#### limitations of the proposed system

A first type of limitation of our systel is related to relation extraction. Indeed, the proposed pipeline does not predict a direct relationship between an adverse event and a drug as defined, for example, in relation extraction tasks in NLP. As a matter of fact, the AE-drug relationship classifier is only trained to categorize meaning units into 2 categories “states a relationship” or “does not state a relationship”. Therefore, if two AEs and two drugs are coexisting in the same meaning unit, the pipeline is not able to separate and identify the possible multiple AE-drug relationships. Meanwhile, due to the relatively reduced length of meaning units (25 tokens on average and max 80–100 tokens) this situation remains very rare and has low impact on the performance.

Another limitation is related to the very assessment of the recall. Indeed, one of the main difficulty in assessing the performance of such systems lies in evaluating the proportion of documents existing in the literature, that are actually missed by the system. As mentioned during the results presentation, this would require the extraction of a test sample with the same distribution of relevant documents available in the literature. Unfortunately, except with a comprehensive work consisting of reviewing hundreds of articles and a strict control of bias during article selection, it is very difficult to get a correct and unbiased estimation of the recall. Instead, we chose to evaluate the recall only within relevant documents at sentence level.

## Conclusion

In this paper, we presented the LiSA approach, a deep learning based pipeline for Adverse Drug Reaction monitoring in the biomedical literature. To our knowledge, our work is the first one to rely on a modular architecture of open-source fine-tuned models and providing access to multilevel outputs (AE/Drug relationship, AE and Drug entities, ADR Seriousness monitoring). We evaluated the performance of the system at two levels a) predictive performance based on a benchmark dataset labeled by medical reviewer and made available for future research and b) user-based performance where ADR monitoring with LiSA is compared with a semi-manual work based on keyword search on PubMed search engine. We have shown that based on LiSA user interface, a medical reviewer is able to retrieve 2.5 times more relevant documents than with a simple semi-manual search. Assisted literature monitoring with deep learning has proved to be a viable an extremely efficient approach to address the current challenges in pharmacovigilance. Future research could move toward assessing relationships across the boundaries of single units of meaning, attempting to combine the benefits of the deep learning described here with traditional language models, which would expand the application areas of the pipeline described here for other pharmacovigilance tasks.

## Data Availability

The data that support the findings of this study are available from the excel file that our research group created as a supplementary material.

## Appendix

**Table:**

Preferred name	Synonyms used when available
Fluticasone Fluticasone furoate	FLUTICASONE FUROATE/ GSK 685 698/ GSK685968/ GSK-685968/ GW685698X/ GW-685698X
PEMBROLIZUMAB	KEYLYNK-010 COMPONENT PEMBROLIZUMAB/ LAMBROLIZUMAB/ MK-3475/ PEMBROLIZUMAB/ PEMBROLIZUMAB COMPONENT OF KEYLYNK-010/ SCH-900475
BAY2327949	
NIVOLUMAB	NIVOLUMAB/ ONO-4538/ MDX-1106/ BMS-986298/ BMS-936558
IPILIMUMAB	BMS-734016/ MDX-CTLA-4/ MDX-101/ MDX-CTLA4/ MDX-010
METAMIZOLE SODIUM	DIPYRONE/ METAMIZOLE SODIUM/ METAMIZOLE SODIUM MONOHYDRATE/ METHAMPYRONE/ NORAMIDOPYRINE METHANESULFONATE SODIUM/ NSC-73205/ SULPYRINE/ SULPYRINE HYDRATE
IFOSFAMIDE	IFOSFAMIDE/ MJF 9325/ MJF-9325/ NSC-109724/ Z4942/ Z-4942/ Ifex/ Ifsofamide/ MITOXANA
MK-8931	
Darboepoetin alfa	
EPOETIN ALFA	
INGENOL MEBUTATE	AGN 204332/ INGENOL MEBUTATE/ PEP005/ PEP-005
Cisplatin	NSC-131558/ TRANSPLATIN/ Cisplatin/ Platinol/ Platinol-AQ
DEBIO 1143	DEBIO 1143/ AT-406/ D-1143/ DEBIO-1143/ IAP INHIBITOR AT-406/ SM-406/ XEVINAPANT
REMDESIVIR	GS 5734/ GS-5734/ REMDESIVIR
RIVAROXABAN	BAY 59-7939/ BAY-59-7939/ JNJ39039039/ JNJ-39039039/ RIVAROXABAN
Atezolizumab	ATEZOLIZUMAB/ Anti-PDL1/ Anti-PD-L1/ MPDL3280A/ MPDL-3280A/ RG7446/ RG-7446/ TECENTRIQ
FINGOLIMOD	Fingolimod/ FINGOLIMOD/ FTY-720/ FINGOLIMOD HYDROCHLORIDE/ FTY720/ FTY-720 HYDROCHLORIDE/ TY720 HYDROCHLORIDE

## Abbreviations

NLP: Natural language processing
NER: Named entity recognition
ADR: Adverse drug reaction
AR: Adverse reaction
SADR: Severe adverse drug reaction
AE: Adverse effect
ADE: Adverse drug effect
SAE: Serious adverse effect

## Footnotes

1 This number was chosen by experts as a good compromise between timeliness of information and the certainty of not missing a relevant signal that might have been reported some time ago.
